# EXPLORING “LIVING WELL” FROM THE PERSPECTIVE OF PEOPLE LIVING WITH ALZHEIMER’S DISEASE AND RELATED DEMENTIAS

**DOI:** 10.1093/geroni/igad104.2651

**Published:** 2023-12-21

**Authors:** Sam Fazio, Lauren Stratton

**Affiliations:** Alzheimer’s Association, Chicago, Illinois, United States; Alzheimer’s Association, Chicago, Illinois, United States

## Abstract

Alzheimer’s disease is a progressive, degenerative disease; however, at the same time individuals living with dementia experience symptoms, they also want to live well with the disease. Although living well with dementia holds different meaning to different people, it generally includes some commonalities, such as maintaining autonomy and independence. To explore the concept of living well with Alzheimer’s disease and related dementia (ADRD), the Alzheimer’s Association gathered data from nearly 100 individuals living with dementia through focus groups and online surveys. Themes from the focus groups were revealed on living well, including being viewed as an individual (person-centered care), staying involved, and receiving multiple types of support to assist their well-being. Open-ended questions from the surveys illustrated additional themes related to living well, as well as on personal meaningfulness of disease modifying therapies including more time with loved ones and opportunities to be part of significant family events. Within these findings, an underlying theme emphasizing the importance of person-centered care and well-being arose. Further discussion will explore these interconnected concepts of living well, person-centered care, and well-being, specifically including the perspectives of individuals living with ADRD. In addition, these concepts can be utilized to tailor resources and support to better support individuals living with ADRD to live well. Lastly, the concept of living well in relation to the emergence of disease modifying therapies and how personal meaningfulness is just as important as clinical meaningfulness will also be explored.

